# Policy Analysis of the Integration of Sports and Medicine against the Backdrop of “Healthy China”: A Qualitative Study Using NVivo

**DOI:** 10.3390/ijerph20032079

**Published:** 2023-01-23

**Authors:** Baihui Wang, Qinqin Lin, Yawei Wang, Shaokai Tang

**Affiliations:** 1 School of Physical Education, Yanshan University, Qinhuangdao 066004, China; 2 School of Public Administration, Yanshan University, Qinhuangdao 066004, China

**Keywords:** Healthy China, sports-medicine integration, NVivo qualitative analysis

## Abstract

In China, the aim of integrating sports and medicine is part of a national health promotion policy. It is important to clarify the relevant policy points, policy practice distribution, and practical tools, as well as to find the weak links in the policy. In the study, there are 34 primary child nodes, 12 secondary child nodes and four parent nodes that were formed. In this study NVivo 11 software was used to analyze the content of 15 national guidelines in terms of integrating sports and medicine. From 2014 to 2021, the policy development of the integration of sports and medicine went through the beginning and growth stages. The evolutionary logic presents an inverse relationship between the policy practice’s duration and the degree of state intervention. In the sequential developmental phases, policy tools were set up in an orderly transition from a single mandatory policy tool to a voluntary or hybrid policy tool, supplemented by essential policy tools. With respect to the policy content, the attention to specific service groups and sports risk assessment is insufficient. In the future, we should actively focus on the division of particular service groups and their service supply, pay closer attention to the social needs and value manifestation of sports risk assessment, and balance the proportion of policy tools in the development of the integration of sports and medicine.

## 1. Introduction

With the ageing of China’s population, the incidence of chronic diseases continues to rise; furthermore, health issues have attracted widespread attention from society. From the advocating of mass sports activities to forming a national fitness system with Chinese characteristics, thereby enhancing physical fitness and health promotion, the state has been guiding the masses via policy support and policy means. It has continuously encouraged awareness of the potential of national fitness and health. In 2016, the Central Committee of the Communist Party of China and the State Council promulgated and implemented the “Healthy China 2030” planning outline, which elevated “Healthy China” to the status of a national strategy. The “Healthy China 2030” plan contained a proposal to strengthen the integration of sports and medicine, as well as non-medical health interventions. This plan was released and put into effect in 2016 by the Central Committee of the Communist Party of China and the State Council. The in-depth integration of sports and healthcare became a key strategy in order to achieve a healthy China and promote the health of all people [1]. The integration of sports and healthcare has become an important task in order to achieve better health in China and to promote the health of all people [2]. Research on the integration policy of sports medicine is mainly based on the importance of policy, foreign policy experience, and local policy practice analysis. Some studies have shown that the change from disease treatment to a health promotion concept is the correct backdrop in which to utilize the establishment of an integration policy; furthermore, the policy-driven strength will affect the development of practice [3]. In addition, the integration policy of sports medicine in China is mostly mentioned in sports and medical health documents; however, no policy document on the integration of sports medicine has been released, and there is a lack of top-level planning and design [4]. Other studies show that foreign policies on the integration of sports and medicine integration pay greater attention to financial security, infrastructure construction, and the evaluation of implementation effects [5]. Generally, the research results of sports medicine integration policies are relatively few, and the research depth is still shallow; furthermore, the policy content has not been properly focused upon. It is, therefore, an urgent priority to analyze the current policy system, consider what kind of theoretical policy system to build, and to fill the research gap on policies for the integrated development of sports and health care. The guidance, supervision, and regulation of policies are essential in order to better promote the integrated development of sports and healthcare. Therefore, in this paper, guided by the concept of health promotion for all and with the help of the qualitative research function of NVivo software, an analysis of the main points is carried out: policy deficiencies and the use of practical tools of sports medicine integration development policies at the national level. Moreover, the aim is to provide scientific opinions and suggestions for the formulation and policy practice of sports medicine integration policies in China for the future.

## 2. Study Design

### 2.1. Data Sources and Selection

In this study, the principles of authority and comprehensiveness in selecting policy documents were followed. Furthermore, by searching the websites of the Central People’s Government of the People’s Republic of China, the State Sports General Administration, the National Health and Wellness Commission, and the database of the Peking University Magic Weapon, we collected and organized policies issued by the central government (or departments that were directly under the central government) in recent years under the themes of “integration of sports and medicine” and “Combination of sports and medicine”. Additionally, “Sports-Medical Integration” and other related policies involving the integration of sports and medicine comprised the final 15 relevant policy documents from 2014 to 2021 that were analyzed (see Table 1).

### 2.2. Research Tools and Methods

This study mainly adopts the qualitative research method of rooted theory, which usually does not study the hypothesis, advocates sorting out and summarizing concepts and categories from the original data, and gradually abstracts into a specific theory through the repeated comparison of materials [6]. Rooted theory was first applied to sociological research, and has gradually emerged in management, information science, physical science and other research fields [7,8,9]. This paper adopts the rooted theory method based on the policy text and cluster analysis functions of NVivo 11 software (QSR International (Americas) Inc, Burlington, MA, USA). We analyzed 15 policy texts related to the integration of sports and medicine released at the national level. In order to count the number of sentences in content with respect to the integration of sports and medicine, we preliminarily established the analysis framework of this study through the word frequency analysis function, interpreted the policy content structure with the coding analysis function, and discovered policy deficiencies with the use of the cluster analysis function.

The coding process in this study was conducted based on a traditional theory research approach. After collecting, organizing, and importing policy texts, all policy content involving the integration of sports and medicine were included in the coding. Through open coding, the extraction of concepts and categories was performed. The policy text information was broken up, and the same, or similar, types were selected via spindle-based coding. After the first stage of open coding was complete, the resulting concepts and categories that were left simplified the content of many primary sources. Additionally, the independent variables in the theoretical structure system gradually appeared. It is necessary to link the different types and to reintegrate the decomposed text in order to further explore the relationship between the categories in the open-coding stage. Selective coding identifies the core classes, further analyzes the logical relationships previously summarized, forms core genera (and verifies the relationships between them), and finally makes the core genera encompass all of the codes [10]. To ensure the comprehensiveness, objectivity, and validity of the coding, we drew on the relevant policy analysis theories and policy research results [11,12] in order to determine the keywords of each core category against the framework of healthcare integration services [13]. In the process, in order to mitigate the influence of subjective judgment on the coding, the coding process was carried out independently by two people in accordance with a pre-established nodal system. After completing the initial round of three-level coding, the coding consistency between the two people was compared, tested, and corrected via calculating the percentage of agreement between them. It was in this manner that the coding results were ultimately determined [14].

### 2.3. Theoretical Basis and Analytical Framework

In this paper we drew on Li Jingyuan’s [15] study on the “connotation and pathways of the integration of sports and medicine” and Chen Xiaohong [16] and colleagues’ study on “the construction of the service framework of the integration of sports and medicine in China in the context of active health”. In addition, we systematically interpreted the connotation, essence, and service content framework of the integration of sports and medicine. Firstly, it is clear that physical activity effectively promotes physical fitness, disease prevention, rehabilitation, and complementary treatment. Secondly, it is shown that medical theories and methods are applied to sports and fitness; in doing this, these theories and methods play a role in sports risk assessment, sports injury prevention, diagnosis, and treatment for the purposes of effectively avoiding sports risks. Again, it is stressed that the integration of sports and medicine is led by the government, with the joint efforts and participation of all social parties, and its purpose is to serve people’s health. Moreover, the government, through doing this, aims to achieve the prevention and control of disease occurrence and development, to reduce medical costs, and to improve quality of life.

The analysis framework of this study was initially set based on the word frequency analysis results as obtained through the use of NVivo software. To highlight the research topic and to filter out interfering information, all the texts related to the integration of sports and medicine were first selected by open coding. The word frequency analysis was conducted for all the readers under the parent nodes. Verbs and dummy words that could not express the policy focus or did not refer to specific policy content were included in the deactivation list, such as “strengthen” “improve” “carry out” and “promote”. The results of the word frequency analysis results are shown in Table 2.

First, in Table 2, the terms “health”, “service”, “sports”, “fitness”, “national”, “hygiene”, “medical”, and other terms appear more frequently. This is in line with the essential characteristics of the integration of sports and medicine, which uses sports as a means by which to apply medical theories and methods to sports and fitness and to serve all people’s health. In addition, the open coding of this study is scientific and feasible around the integration of sports and medicine. Second, the frequency of specific service-related terms such as “nutrition”, “monitor”, “chronic disease”, “activity”, “guidance”, “prevention and treatment”, and other specific service-related terms also appeared more frequently. This indicates that the country is beginning to pay attention to national physical fitness and chronic diseases and is beginning to recognize the importance of exercise. Again, “standard”, “mode”, “population”, “environment”, “department”, “community”, and other terms have also begun to appear more frequently. In addition, “data”, “information”, “technology”, and other words representing safeguards have also begun to appear more frequently. Therefore, based on the results of the word frequency analysis, we addressed the diversified characteristics of the content of the integration of sports and medicine. However, we considered the differences in the needs of different people for the purposes of integrating sports and medicine. As such, in this study, we analyzed four aspects: task orientation, capacity building, assessment and evaluation, and service content.

## 3. Research Results

### 3.1. Policy Practice Distribution

**Figure 1:**
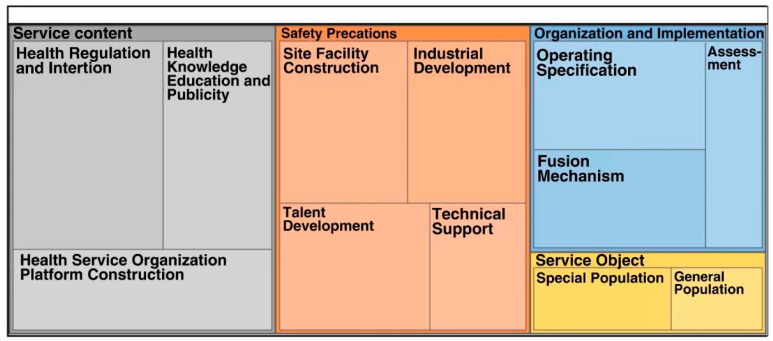
Policy and practice distribution map of the sports and medical integration service.

In this study, 15 policy texts were read and analyzed in an open coding process, resulting in 235 reference points. The spindle coding process summarized all reference points, forming 34 primary sub-nodes and 12 s-nodes. For example, physical fitness monitoring, exercise prescription, and national health activity status surveys are summarized as health regulation and intervention. The selective coding process analyzes the connection between the second-level sub-nodes. It then translates them into four sub-nodes: “service population”, “service content”, “guarantee measures”, and “organization and implementation”. The existence of intrinsically linked tree nodes interprets the content focus and hierarchy of health promotion policies regarding integrating physical medicine. Fifteen policy texts describe the service content, safeguards, and organization implementation of ageing health work, whereas only six subdivide the policies’ specific target groups. The numbers of reference points for the four parent nodes are 89 service contents, 25 service objects, 71 safeguard precautions, and 50 organization implementations. The distribution of specific policies and practices is shown in Figure 1.

In order to check the accuracy of coding and to avoid subjectivity, coding reliability was tested after r-coding the sample literature. The coding agreement between the two coders was tested by calculating the percentage of understanding between them. The calculation formula is the percentage of agreement = [number of mutually agreed codes/(number of mutually agreed codes + number of mutually disagreed codes)] × 00%. The inter-investigator reliability should be greater than 70% [17]. The results show that the number of mutually agreed principles was 207, and that the number of mutually disagreed codes was 28. When substituting the formula with these results, the percentage of agreement = 207/(207 + 28) × 100% ≈ 88.09%. As this is higher than the 70% inter-investigator reliability threshold as mentioned above, we determined the coding reliability to be good.

### 3.2. Analysis of the Main Points of the Policy of Integration of Sports and Medicine

#### 3.2.1. Analysis of the Main Points of the Policy Service Content with respect to the Integration of Sports and Medicine

**Figure 2:**
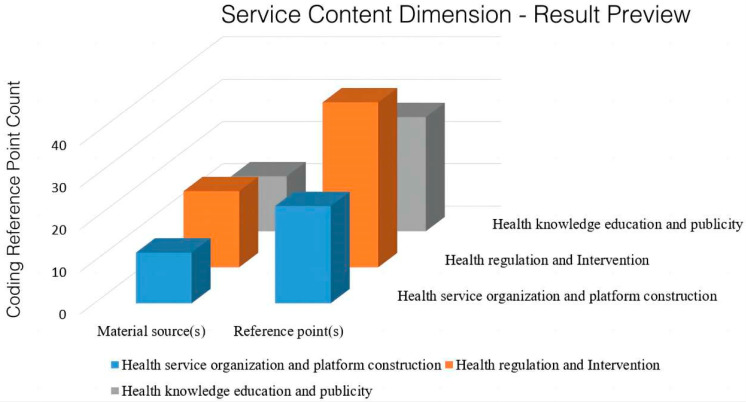
Distribution of reference points for the service content of the integrated medical policy.

The distribution of reference points for the service content of national-level policies on the integration of sports and medicine is shown in Figure 2. A total of 89 reference points related to service content were found in the 15 policy texts, including three secondary sub-nodes of health service institutions and platform construction, health supervision and intervention, and health knowledge education and publicity. The service content of the integration of sports and medicine encompasses three levels of ideology: practical action and infrastructure supply; the reflecting of both active and passive; and the strengthening of health intervention. The provision of health service institutions and platforms, as well as health supervision and intervention services, tend to be passive measures. The supply of services from the sports and medical sectors is highlighted in the policy, i.e., health guidance, knowledge promotion, etc.; these tend to be the dynamic behaviors of the masses that enhance their autonomy and improve their health through health guidance and knowledge learning. Other studies show that Singapore, Australia and other countries have proposed the promotion of the active participation of fitness programs at the national level with the purpose of popularizing fitness knowledge to residents and to cultivate fitness habits [18,19].

#### 3.2.2. Analysis of the Main Points of the Population Served by the Policy of Integration of Sports and Medicine

**Figure 3:**
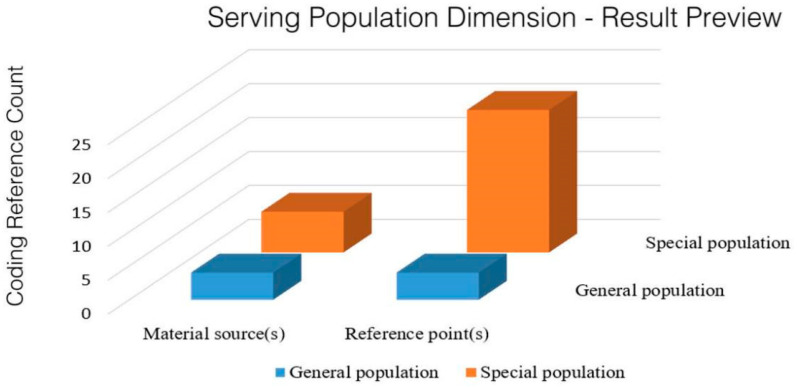
Distribution of reference points for the population served by the integration of the sports and medicine policy.

Figure 3 shows the distribution of reference points for the population served by the policy of the integration of sports and medicine at the national level. Twenty-five reference points of the refined classification of the population were obtained in 15 policy texts, including in the special and general populations. The division of the people reflects the emphasis on the different health needs of the different social groups, which is in line with China’s people-oriented development concept. Special groups are composed of adolescents, the elderly, and chronically ill groups. It should be noted that the number of reference points in the above group classifications is relatively small.

#### 3.2.3. Analysis of the Main Points of the Policy Safeguards for the Integration of Sports and Medicine

**Figure 4:**
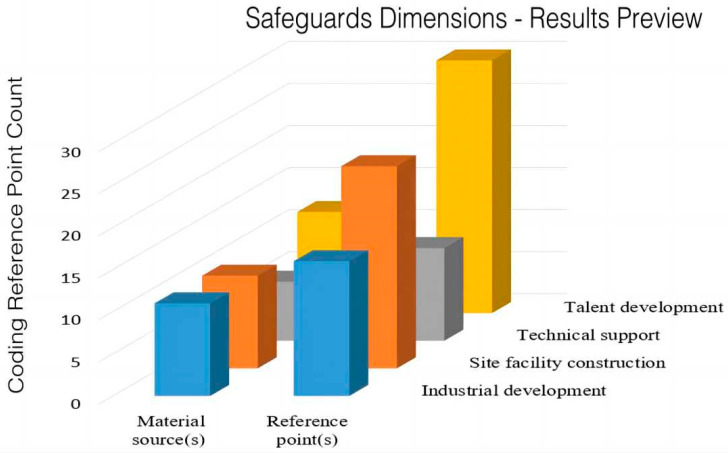
Distribution of reference points for safeguard measures for the integration of sports and medicine.

Figure 4 shows the distribution of reference points for the safeguards of sports medicine integration policy at the national level. A total of 71 reference nodes are related to the safeguards in 15 policy texts, including four aspects of industrial development, site facility construction, technical support, and talent development. Among them, the number of site facility construction and talent training reference points is high, indicating that the policy of sports medicine integration pays more attention to grass-roots capacity building. Additionally, most of the policies pay attention to the construction of hardware facilities, encouraging the opening of sports venues, and improving public sports facilities, and the effect is obvious. Studies have shown that in order to solve the problem of resource shortage of sports facilities, regular sports activities should be simply treated, and people are encouraged to actively participate in physical exercise [20].

#### 3.2.4. Analysis of the Main Points of the Organization and Implementation of the Policy of Integration of Sports and Medicine

**Figure 5:**
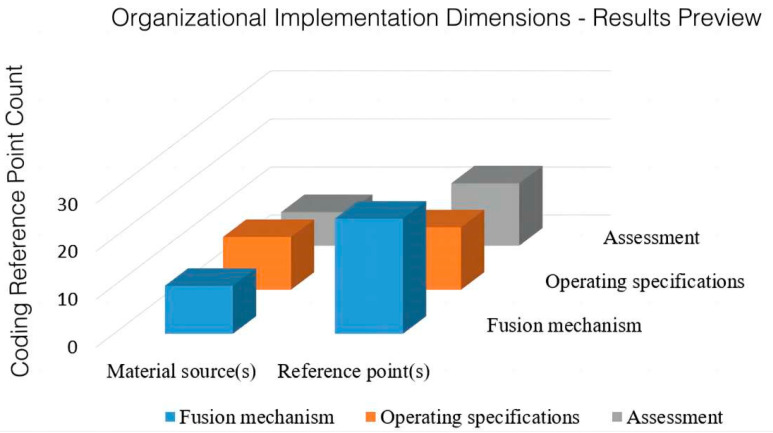
Distribution of reference points for the organization and implementation of the integration of sports and medicine policy.

Figure 5 shows the distribution of reference points related to the organization and implementation of the policies promulgated at the national level regarding the integration of sports and medicine. There are 50 reference points regarding organization and implementation in 15 policy texts, specifically including the three aspects of operating specifications, assessment, and fusion mechanisms. The content of the fusion mechanism is the most involved; its content is based on inter-departmental structural synergy, therefore advocating interdepartmental institutional innovation and institutional integration. For example, the China Institute of Sport Science takes the lead in establishing the Research Center for the Promotion and Innovation of Sports-Medicine Integration. The Health Promotion Committee is set in the medical and health system. The development of assessment, evaluation, and operational norms reflects the guidance of goals and the willingness to assess. This is found in the inclusion of scientific national fitness guidance services in the measurement system of creating model cities and counties for the purposes of national sports and fitness, as well as in the inclusion of health promotion and education in the context of governmental goal assessment, etc., in order to provide macro-planning for practical actions.

## 4. Coding Cluster Analysis of Integration of Sports and Medicine Policy Text

**Figure 6:**
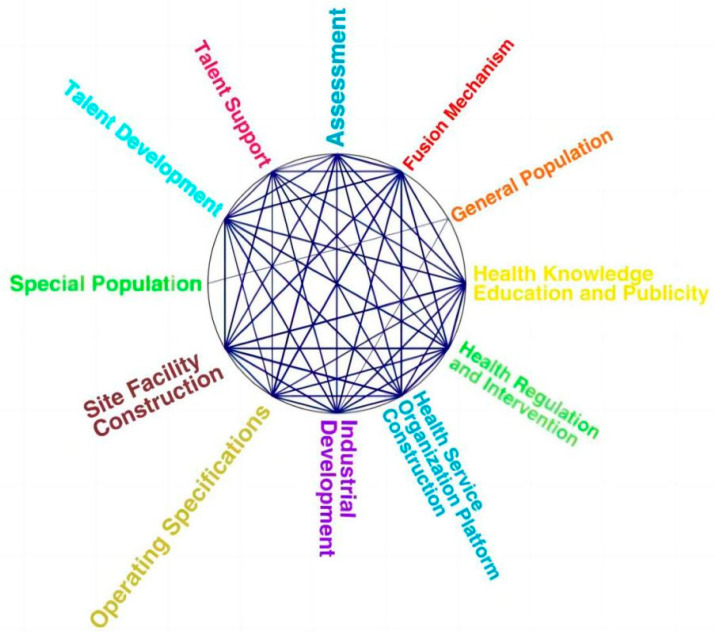
Clustering analysis results based on coding similarity.

The identification of policy issues in this study is based on two aspects. On the one hand, the clustering analysis results from the NVivo software were used to observe the coding nodes of the content of the policies enacted at the national level regarding the integration of sports and medicine and to identify the weak points in the guidelines. On the other hand, the above coding framework was compared with the connotations regarding the integration of sports and medicine, as well as the theoretical framework for the integration of sports and medical services. In doing this, the existing policies were found to be insufficient. We used the Pearson correlation coefficient as a metric to cluster the secondary sub-nodes under the four parent nodes. When the coding similarity was between 0.5 and 1, a clustering analysis result that could reflect the policy’s blind areas was obtained (see Figure 6). In Figure 6, the policy focus and weakness are indicated by the density of the lines. The denser lines suggest that the coding of the region is emphasized in the policy text, while the sparser lines indicate that the coding of the area is neglected in the policy text.

### 4.1. Insufficient Attention to Specific Service Populations

As shown in Figure 6, the thin and sparse lines between the special and general populations, as well as the other nodes in the specific service population, may be the weak links of the current healthcare integration policy. Among the service populations of the policies, special populations such as the elderly and the chronically ill are given more attention than the general population. They are commonly mentioned in several approaches, but their attention is very low compared with the secondary nodes in other parent nodes. Targeted policies for specific service groups have not received widespread attention, and healthcare integration services’ social needs and value functions have not fully manifested. The reason for this may be that the policy of sports medicine integration is still in the planning and document development stage. This, therefore, means that it cannot provide appropriate guidance and interventions for the key population groups in the development process due to the low association between the sports and medical sectors, as well as due to the lack of professional talent [21]. Another study found that the policy of sports medicine integration not only focuses on chronic disease patients in terms of prevention and rehabilitation, but also classifies chronic disease patients with different conditions in specific disease groups [22]. For this reason, the policy development process should focus on organizing particular service groups and gradually expanding the scope of the integration of sports and medical services.

### 4.2. Lack of Attention to Sports Risk Assessment Issues

The framework for integrating sports medicine services is guided by sports medicine theory and is based on the safety, effectiveness, and sustainability of health promotion exercises. However, the analysis and coding results of 15 policy texts showed that the assessment of exercise risk in most policy texts was covered in the content of physical fitness monitoring and fitness guidance without specific subdivision of the content of exercise risk assessment, which may be a shortcoming of the current sports medicine integration policy. The reason for this may be related to the fact that the framework of sports medicine integration services has not yet been widely popularized nationwide, the concept of sports medicine integration is relatively weak among the supply and audience subjects, and most people’s health philosophy is still based on “light exercise, heavy medical care” [15]. The concept of changing from “treatment has been sick” to “preventive treatment of sick” has not been fully recognized. Some studies have shown that the health literacy of Chinese residents has steadily improved from 2012 to 2020, and that, therefore, the target of “Healthy China 2030” has been achieved [23]. Another study shows that the prevention and control of chronic diseases based on “medical intervention” has been changed to “behavioral improvement + medical intervention”. In addition, studies have shown that it is an inevitable choice for chronic disease prevention and control to switch to a balanced development approach of “behavioral improvement + medical intervention” [24]. In the process of the integration of health and medicine in western countries, while encouraging citizens to participate in fitness, they have also established an evaluation mechanism to move the health threshold forward [25]. For this reason, we should focus on improving the policies related to exercise risk assessment in order to provide a safety guarantee for people’s independent health behavior.

## 5. Policy Evolution and Use of Practical Tools

### 5.1. Policy Evolution

Since the 1990s, when the Sports Law and the Outline of the National Fitness Plan were proposed in order to carry out physical fitness monitoring—sports and medical care integration has been gradually mentioned in sports and medical policies. On 20 October 2014, the State Council issued the “Opinions on Accelerating the Development of Sports Industry and Promoting Sports Consumption”, which proposed to carry out the integration of sports and recreation. Additionally, the aforementioned “sports prescription” was a landmark term for the integration of medicine, marking the first time that a policy on the integration of medicine and sports was introduced at the national level. The “Guiding Opinions on Accelerating the Development of Lifestyle Service Industry and Promoting the Upgrading of Consumption Structure” issued in 2015 also mentioned the integration of sports and recreation. The year 2016 saw the successful introduction of policies such as the National Fitness Plan (2016–2020) and the Outline of the Healthy China 2030 Plan (from now on referred to as the Outline), which was proposed to promote the physical activity of key populations by the strengthening of the integration of sports, medicine, and non-medical health interventions; from this period onwards, articles on the analysis of the policy of integration of sports and treatment have been presented one after another.

Based on the policy context, we analyzed the degree of adaptation of the basic public services of sports medicine integration in society. As the government provides basic public services, scholars have explored the dilemmas and practical paths of developing sports medicine integration services by analyzing policy texts and case studies [26,27]. Other studies, based on the policy text itself, have focused on optimizing the policy content, exploring the development strategies of sports-medical integration under the guidance of the policy, and using the policy as a guide in order to create an outlook on the future development of sports-medical integration [17,28,29].

**Figure 7:**
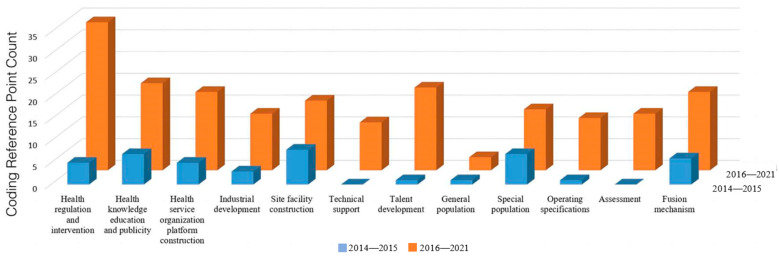
The distribution of the number of stages of the integration of sports and medicine policy.

Research on the policy of sports and medical integration has been carried out in China for a relatively short period. Its evolutionary process can be divided into two stages through data analysis, as shown in Figure 7. The period 2014–2015 saw China’s sports medicine integration policy in its infancy stage, which was mainly reflected in a macro-government plan as a blueprint, together with government investment in an infrastructure project, direct subsidies for the supply of public services for health promotion, and the direct regulation of the public service market. During the period 2016 to 2021, the integration of sports and medicine policy was in the development stage, which is mainly reflected in the government’s guiding opinions, financial policy support for service providers, etc., together with the introduction of social capital, demonstration, and leadership in the pilot areas for the integration of sports and medicine, as well as preferential policies for various service-supplying subjects. During this period, incentive- and guidance-based policies for the purposes of integrating sports and medicine were initially established. On the one hand, these fostered the independent participation of social service subjects. On the other hand, they maintained the level of service supply of compulsory forces in basic key areas, keeping the market reluctant to become involved in the strong collection for key regions, while adopting encouraging policies in order to attract the active participation of social forces.

### 5.2. Practice Tool Usage

**Figure 8:**
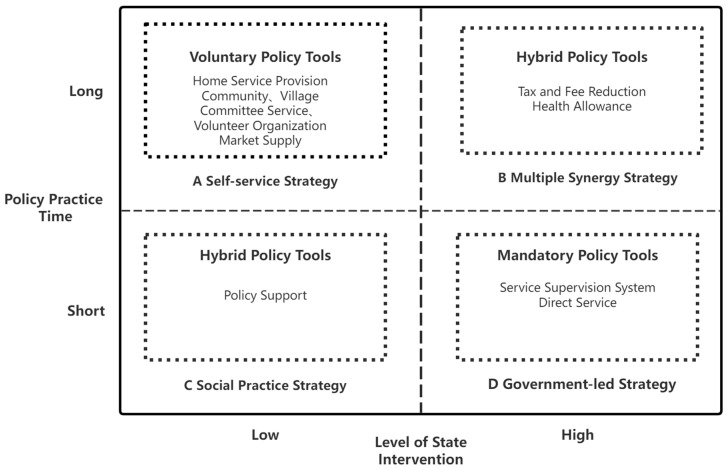
Practical tools of sports medicine integration policy.

The evolution stages of the policy for corporate-health integration mainly present an orderly transition from a coercive strategy of adopting a single coercive policy tool to a hybrid system with resource or hybrid policy tools as the main tool and coercive policy tools as a supplement, as shown in Figure 8.

In the initial stage, mandatory policy tools are used more often in order to provide strong support for the efficient development of the integration of sports and medicine and to provide a strong institutional environment for the subsequent development of sports and medicine. In the development stage, China’s policy on healthcare integration began to include voluntary and hybrid policy tools, with macroscopic government programs used in order to complement the developing social market forces for the construction of healthcare integration services. Overall, the evolutionary logic of the integration of sports and medicine policy shows an inverse relationship between the length of policy practice and the degree of state intervention.

## 6. Conclusions

With the help of NVivo 11 software, this study analyzed the policy texts on the integration of sports and medicine from the websites of the Central People’s Government of the People’s Republic of China, the General Administration of Sport of China, the National Health Commission, and other websites, and the magic database of Peking University. Through the construction of the analytical framework, policy content structure and the discovery of policy deficiencies, we explored the integration policy of sports and medical care in China, analyzed its qualitative content, and achieved research results. The following are the shortcomings and future prospects of this study.

### 6.1. Research Limitations

First of all, the policy texts selected for this study are from the website of the Central People’s Government of the People’s Republic of China, the General Administration of Sport of China, the National Health Commission, and the magic weapon database of Peking University. Regarding its use in the Nvivo11 software, the number of this study is not dominant. Secondly, the policy text is not a specific policy of the integration of sports and medicine, so there is a lack of specific example policy text analysis in the research content, which is also a problem in the development of the integration policy of sports and medicine in China.

### 6.2. Future Prospects of Policies of the Integration of Sports and Medicine in China

#### Active Attention to the Division of Specific Service Populations and Service Supply Issues

In the “Healthy China 2030” Plan, improvement of the physical quality of the whole population is proposed by broadly carrying out national fitness campaigns, strengthening the integration of sports, medicine, and non-medical health interventions, thereby promoting the intervention of physical activities for different groups of people, and therefore establishing an integrated health service model of prevention, rehabilitation, and treatment. Existing studies have shown that significant differences in the total demand rate for education and physical medicine integration services should be carried out according to the demand degree of different age groups and social groups [30]. First, education and publicity regarding the knowledge of chronic disease rehabilitation for the elderly population should be strengthened in order to popularize fitness modalities that can be performed independently with lower risks. At the same time, special rescue, accompanying medical treatments, and scientific fitness service mechanisms should be established for the elderly group. Second, health education for the younger age groups should be strengthened, and the health knowledge assessment system should be improved in order to draw the attention of this group towards health and to play the role of a health warning system. Third, the scientificity and effectiveness of the occupational group should be strengthened; furthermore, smart sports should be vigorously developed to use the internet, Big Data, and other technologies in order to provide scientific, effective, and convenient health promotion channels.

### 6.3. Valuing the Social Needs of Sport Risk Assessment

With the promotion of sports medicine integration services, the concept of sports as good medicine has gained popularity, and people’s awareness of sports has gradually increased; moreover, issues such as health risks and injury risks in sports have become increasingly prominent. Health risks, which can also be referred to as sports injury risks [31], refer to the potential problems of pre-existing diseases or risk factors in sports with acute onset and serious consequences. In contrast, sports injuries refer to muscle, joint, and bone injuries caused during sports. In addition to their health conditions, the predisposition also includes the adequacy of warm-up preparation before exercise, the appropriateness of sports attire, the safety of the venue equipment, and the sports environment. Therefore, given the above problems, it is recommended that the risk of the sport assessment test should be opened up in regard to the integration of sports and medical services; furthermore, corresponding sports advice and risk tips should be given in the process, such that basic tips and risk tips should be given in the process of basic physical fitness monitoring in response to the physical fitness test results. Moreover, it is advisable to introduce the methods of Big Data and other technical analysis means in order to provide efficient, scientific, and accurate sports risk assessment. In addition, fitness facilities and environment constructions should be strengthened in order to provide basic protection for the public when engaging in physical activity.

### 6.4. Focus on Balancing the Proportion of Policy Tools Used

As the main vehicle of the policy text, the government’s choice of policy instruments has an important impact on achieving policy goals during the policy implementation phase. The distribution of policy tools, in practice, for integrating sports and medicine shows that the length of policy practice is inversely proportional to the degree of state intervention. Existing studies show that the ratio of the use of supply- and environment-based policy tools in China’s corporate sports medicine integration policies accounts for 80%. In comparison, demand-based policy tools only account for 20% [32]. For this reason, it is recommended that the use of demand-based policy tools should be strengthened in future policies, and policy formulation should focus on the synergy of multiple subjects, decentralizing government authority, and encouraging, supporting, and guiding more social organizations and social forces in order to participate in the practice of physical and medical integration services. Furthermore, it is advised that we should strengthen the research on the social situation of the integration of sports and medical services, as well as clarify the service needs of grassroots processes. In addition, it would be wise to develop policy contents with different details about sports and medical integration, as well as details for other regions and different service subjects.

## Figures and Tables

**Table 1:** Policies related to sports medicine integration at the national level.

Date of Promulgation	Document Name	Policy Sources
20 October 2014	Some Opinions on Accelerating the Development of Sports Industry and Promoting Sports Consumption	General Office of the State Council
22 November 2015	Guiding Opinions on Accelerating the Development of Life Service Industry and Promoting the Upgrade of Consumption Structure	General Office of the State Council
25 October 2016	Outline of “Healthy China 2030” Plan	The CPC Central Committee and the State Council
28 October 2016	Guiding Opinions on Accelerating the Development of Fitness and Leisure Industry	General Office of the State Council
16 November 2016	Guiding Opinions on Strengthening Health Promotion and Education	Ministry of Education, etc.
22 January 2017	Notice on Printing and Distributing China’s Medium and Long term Plan for the Prevention and Control of Chronic Diseases (2017–2025)	General Office of the State Council
27 April 2017	Notice on Issuing the Action Plan for Healthy Lifestyle for All (2017–2025)	National Health Commission, etc.
16 May 2017	Opinions on Supporting Social Forces to Provide Multilevel and Diversified Medical Services	General Office of the State Council
30 June 2017	Notice on Issuing the National Nutrition Plan (2017–2030)	General Office of the State Council
15 July 2019	Opinions on Implementing the Action of Healthy China	General Office of the State Council
2 September 2019	Notice on Printing and Distributing the Outline of Building a Sports Powerful Country	General Office of the State Council
18 September 2019	Opinions on Promoting National Fitness and Sports Consumption to Promote the High Quality Development of Sports Industry	General Office of the State Council
30 September 2019	Notice on the Action Plan for Promoting the High Quality Development of Health Industry (2019–2022)	National Development and Reform Commission, Ministry of Education, etc.
6 December 2019	Opinions on Promoting the Development of “Internet Social Services”	National Development and Reform Commission, Ministry of Education, etc.
13 October 2021	Circular of The State Council of The People’s Republic of China on Several Opinions on Promoting The Living Service Industry to Improve The People’s Quality of Life	National Development and Reform Commission

**Table 2:** Word frequency analysis results of related nodes with respect to the integration of sports and medicine.

High-Frequency Node	Number of Occurrences	High-Frequency Node	Number of Occurrences	High-Frequency Node	Number of Occurrences
health	708	mechanism	98	combination	60
service	242	chronic disease	96	data	59
sports	224	activity	93	knowledge	58
fitness	197	constitution	92	technology	57
national	128	guidance	89	information	56
work	123	get some action	84	disease	54
society	123	science	80	department	54
hygiene	121	the masses	79	community	53
sports	118	standard	78	organization	53
nutrition	116	system	76	propaganda	51
education	110	mode	71	psychology	50
country	107	crowd	69	recovery	49
medical	105	environment	65	major	48
life	105	physical exercise	62	mechanism	48
monitor	101	prevention and treatment	62	family	44

## Data Availability

The data used to support the findings of this study are available from the corresponding author upon request.
